# Ocular Distribution of Brimonidine and Brinzolamide after Topical Instillation of a 0.1% Brimonidine Tartrate and 1% Brinzolamide Fixed-Combination Ophthalmic Suspension: An Interventional Study

**DOI:** 10.3390/jcm12134175

**Published:** 2023-06-21

**Authors:** Yusuke Orii, Eriko Kunikane, Yutaka Yamada, Masakazu Morioka, Kentaro Iwasaki, Shogo Arimura, Akemi Mizuno, Masaru Inatani

**Affiliations:** 1 Department of Ophthalmology, Faculty of Medical Sciences, University of Fukui, Fukui 910-1193, Japan; 2 Senju Pharmaceutical Co., Ltd., Osaka 541-0048, Japan

**Keywords:** brimonidine, brinzolamide, fixed-combination ophthalmic suspension, vitreous humors, aqueous humors

## Abstract

Purpose: To evaluate the concentrations of brimonidine and brinzolamide in the vitreous and aqueous humor after instillation of a 0.1% brimonidine tartrate and 1% brinzolamide fixed-combination ophthalmic suspension. Methods: The present investigation involved patients with macular holes or idiopathic epiretinal membranes who were planning to undergo vitrectomy. One week prior to surgery, the patients received twice-daily topical treatment with 0.1% brimonidine tartrate and 1% brinzolamide fixed-combination ophthalmic suspension. Before vitrectomy, vitreous and aqueous humor samples were collected, and the mean concentrations of brimonidine and brinzolamide were determined through liquid chromatography-tandem spectrometry. Results: Ten eyes (nine phakic and one pseudophakic eyes; 10 patients) were examined. The concentration of brimonidine in vitreous and aqueous humor samples was 5.02 ± 2.24 and 559 ± 670 nM, respectively. The concentration of brimonidine in the vitreous humor, which is needed to activate α2 receptors, was >2 nM in all patients. The concentration of brinzolamide was 8.96 ± 4.65 and 1100 ± 813 nM, respectively. However, there was no significant correlation between the concentrations of brimonidine in the vitreous and aqueous humor samples. Conclusions: Sufficient concentrations of brimonidine were detected in all vitreous samples. The dissociated correlation of the drug concentrations between aqueous and vitreous humors implies the possibility of another pathway to vitreous humor, different from the pathway to aqueous humor.

## 1. Introduction

Glaucoma is the most common cause of irreversible visual loss in developed countries. Lowering intraocular pressure (IOP) is the most critical strategy for preventing optic nerve damage in glaucoma patients. Various randomized controlled clinical trials and related studies support the strategy that IOP lowering reduces both the onset and progression of glaucoma [1,2,3]. IOP-lowering treatments are classified into three procedures: medical treatment, laser therapy, and surgery. Among the three procedures, medical treatment is commonly used to lower IOP in patients with open-angle glaucoma. Although medical treatments consist of eye drops and oral medications, eye drops are the major medical treatment for glaucoma patients because of their efficiency and tolerance. Currently, various types of IOP-lowering antiglaucoma ophthalmic solutions are available [4,5]. These IOP-lowering antiglaucoma ophthalmic solutions were classified into eight categories: prostanoid receptor-related drugs [6], ß-blockers [7], carbonic anhydrase inhibitors [8], α2 adrenergic agonists [9], rho-kinase inhibitors [10], parasympathomimetic drugs [11], α1 blockers [12], and ion channel openers [13,14]. Among these categories, prostanoid receptor-related drug monotherapy is most commonly administered for the initial treatment of chronic open-angle glaucoma [15]. A randomized clinical trial using latanoprost, a prostanoid receptor-related drug, and the placebo has shown that once-daily latanoprost instillation attenuates visual field progression for glaucomatous eyes in 2 years via its IOP-lowering effect [16]. If further IOP reduction is required to prevent optic nerve damage, an additional eye drop is used for patients with glaucoma previously treated with monotherapy. A recent clinical trial reported that the combination of multiple IOP-lowering drugs with laser treatment was more effective in attenuating the progression of visual field loss than drug monotherapy [17,18]. In developed countries, most patients with glaucoma are treated with multiple types of antiglaucoma ophthalmic solutions [19]. However, half of the patients with glaucoma receiving topical antiglaucoma medications have ocular surface disease [20]. Because of increased exposure to preservatives, multidose topical instillations are associated with a high incidence of corneal epithelial damage in patients with glaucoma [21,22]. The use of multiple topical instillations results in the deterioration of adherence to medical treatment in patients with glaucoma [23]. Thus, despite the intent of further IOP reduction, this therapeutic approach is frequently associated with negative effects on the quality of vision in patients.

Medical treatment with a fixed-combination ophthalmic solution improves adherence and prevents ocular surface disease versus separate instillations of ophthalmic solutions [24]. In Japan, a fixed-combination ophthalmic suspension containing 0.1% brimonidine tartrate and 1% brinzolamide (Ailamide^®^; Senju Pharmaceutical Co., Ltd., Osaka, Japan) recently became available for the treatment of glaucoma and ocular hypertension. The fixed-combination suspension containing 0.2% brimonidine tartrate and 1% brinzolamide (Simbrinza^®^, Allergan, Dublin, Ireland) in the USA and countries in the European Union was the first fixed-combination drug to not contain a ß-blocker [25]. These combination drugs include brimonidine, a highly selective α2 adrenergic agonist that reduces IOP by suppressing the production of aqueous humor and promoting uveoscleral outflow [26,27]. Numerous in vivo and in vitro studies demonstrated that, apart from its IOP-lowering effects, brimonidine exerts potential neuroprotective effects on retinal ganglion cells (RGCs) [28,29,30]. In randomized clinical trials involving patients with open-angle glaucoma, 0.2% and 0.1% brimonidine tartrate ophthalmic solutions reduced the progression of visual field loss versus 0.5% timolol malate ophthalmic solution [31,32]. To perform its neuroprotective function in the RGCs of patients with glaucoma, an adequate amount of brimonidine in the ophthalmic solution should be present in the vitreous humor. Several studies have investigated the concentration of brimonidine in human vitreous samples after topical treatment with 0.1%, 0.15%, or 0.2% brimonidine tartrate ophthalmic solutions [33,34,35]. Following these instillations, most vitreous samples contained a concentration of brimonidine > 2 nM, which is required for the activation of α2 adrenergic receptors in neuronal cells [36].

The efficiency of drug delivery into ocular tissues is influenced by differences in the preservatives used, pH, and viscosity between the fixed-combination and single agents, as well as interactions between components. Our previous study using a 0.1% brimonidine tartrate and 0.5% timolol fixed-combination ophthalmic solution revealed that 63% of vitreous samples contained a concentration of brimonidine > 2 nM [37]. The brimonidine tartrate ophthalmic solution was homogenous. However, the brimonidine tartrate and brinzolamide fixed-combination drug is a suspension due to the composition of brinzolamide [38]. However, the effects of differences in formulations on the pharmacokinetics of brimonidine in the human vitreous remain unknown. Therefore, we examined the concentration of brimonidine in human vitreous humor following the instillation of a 0.1% brimonidine tartrate and 1% brinzolamide fixed-combination ophthalmic suspension.

## 2. Materials and Methods

### 2.1. Patient Selection

The University of Fukui Certified Review Board approved this single-arm open-label interventional trial (approval code: CRB5180014, approval date: 19 October 2020) and adhered to the principles of the Declaration of Helsinki. All participants were informed of the protocol and any potential risks and advantages of the therapies prior to enrolment. All patients provided written informed consent. The Japan Registry of Clinical Trials received registration for this study (jRCT, ID jRCTs051200089; date of access and registration, 25 November 2020).

This investigation involved adult patients (age: ≥20 years) who planned to undergo pars plana vitrectomy for the treatment of macular holes or idiopathic epiretinal membranes between November 2020 and June 2021. The exclusion criteria were as follows: (1) uveitis; (2) vitreous hemorrhage; (3) proliferative diabetic retinopathy; (4) corneal epithelial disorder; (5) a history of allergic reaction to an α2 stimulant or carbonic anhydrase inhibitor; and (6) difficulty in the instillation of ophthalmic solutions.

### 2.2. Sample Collection

Patient characteristics such as sex, lens status (phakia/pseudophakia), age, slit lamp examinations of the ocular surface including palpebral and bulbar conjunctivas and cornea, fundus retinoscopy, and optical coherence tomography for macular holes or idiopathic epiretinal membranes were examined when they were recruited in this study.

The samples were collected as previously described [33]. Briefly, the patients received twice-daily topical instillation of Ailamide^®^ (Senju Pharmaceutical Co., Ltd.), a fixed-combination ophthalmic suspension containing 0.1% brimonidine tartrate, and 1% brinzolamide for 1 week before surgery. On the day of pars plana vitrectomy, ophthalmic suspension was applied at 8:00 a.m. within 2 h prior to surgery. The patients were requested to record their treatment adherence for 1 week using self-check sheets. Patients with an adherence rate of <75% were excluded from the analysis.

As a preoperative medication to prevent postoperative infection, levofloxacin ophthalmic solution 1.5% (Rohto-nitten Co., Ltd. Nagoya, Japan) was also administered for 3 days before surgery. We instructed the patients to allow at least a 10 min interval between multiple instillations.

Standard four-port vitrectomy was performed under retrobulbar anesthesia. Collection of vitreous humor (500 μL) and aqueous humor (100 μL) samples was performed from the anterior chamber and vitreous cavity, respectively. For the avoidance of sample dilution, the infusion line was transiently blocked until the completion of sample collection from the vitreous fluid. A 25 G vitreous cutter pointed toward the optic disc was used for the collection of vitreous humor samples from the area of the retina and optic disc. The samples were placed in Eppendorf tubes and stored at −80 °C.

### 2.3. Sample Size

In our previous investigation of a 0.1% brimonidine tartrate and 0.5% timolol fixed-combination ophthalmic solution (Aibeta^®^; Senju Pharmaceutical Co., Ltd.), the sample size was 8 patients [37]. The University of Fukui Certified Review Board approved the study design to minimize the risk of adverse events to patients because the instillation of Ailamide^®^ (Senju Pharmaceutical Co., Ltd.) had no indication for the treatment of patients with macular holes or idiopathic epiretinal membranes planning to undergo vitrectomy. The sample size of this study was also limited to 10 patients. The sample size was applied to the present trial, including those who withdrew from the study or did not follow the study protocol.

### 2.4. Drug Concentration Measurement

Within 1 month after surgery, the concentrations of brimonidine and brinzolamide in the samples were quantitatively assessed in an independent bioanalytical facility through liquid chromatography and tandem mass spectrometry (CMIC Pharma Science Co., Ltd., Hokuto, Japan). The analysis was performed using a Triple Quad5500 (AB Sciex Pte. Ltd., Framingham, MA, USA) and a Nexera Ultra High-Performance Liquid Chromatography system (Shimadzu Corporation, Kyoto, Japan). Gradient chromatography was carried out using a Kinetex EVO C18 column (inner diameter: 2.1 mm, length: 150 mm, and particle size: 5 μm; Phenomenex Inc., Torrance, CA, USA). 5-Chloro-6-(2-imidazolidinylideneamino) quinoxaline was utilized as the internal standard. The mobile phase consisted of 10 mM ammonium hydrogencarbonate buffer (pH 10.0) and methanol at a flow rate of 0.5 mL/min. Brimonidine, brinzolamide, and the internal standard were examined in the positive ionization mode based on the following multiple reaction monitoring transitions: 292/212 (brimonidine); 384/136 (brinzolamide); and 248/205 (internal standard).

### 2.5. Primary Outcomes

The primary outcomes were as follows: (1) average concentration of brimonidine in the vitreous and aqueous humor samples; (2) percentage of patients with a concentration of brimonidine in the vitreous humor > 2 nM; and (3) correlation between the concentrations of brimonidine in vitreous and aqueous humor samples.

### 2.6. Secondary Outcomes

The secondary outcomes were as follows: (1) concentrations of brinzolamide in vitreous and aqueous humor samples; (2) relationship between the concentrations of brimonidine and brinzolamide in vitreous and aqueous humor samples; (3) changes in IOP after drug instillation; and (4) best-corrected visual acuity (BCVA) prior to and following surgery. To reduce patient distress, a non-contact tonometer (Nidek, Nagoya, Japan) was utilized for the measurement of IOPs in patients with macular holes or idiopathic epiretinal membranes without glaucoma.

The safety of the treatment was continuously monitored throughout the study. Ten to twenty percent of individuals with long-term brimonidine administration encounter allergic conjunctivitis [39]. Other ocular side effects include ocular itching and burning sensation, eye redness, dryness, and corneal opacity [40,41]. General systemic complications are central depression, somnolence, headache, dizziness, vomiting, nausea, and fatigue [42]. We checked the association with ocular side effects using a slit-lamp microscope and instructed the patients to inform our facility immediately if any systemic symptoms appeared.

### 2.7. Statistical Analysis

Statistical analysis was conducted using JMP 15 software (SAS Institute, Inc., Cary, NC, USA). Data are presented as the mean ± standard deviation. Ordinary least-squares regression analysis was used to assess the relationship between the concentrations of brimonidine and brinzolamide in the vitreous and aqueous humor samples. Changes in IOP after treatment were examined using a paired sample *t*-test. For all tests, the level of statistical significance was set at *p* < 0.05.

The patients, intervention providers, or outcome assessors were not blinded to the procedure.

## 3. Results

### 3.1. Patient Characteristics

**Figure 1:**
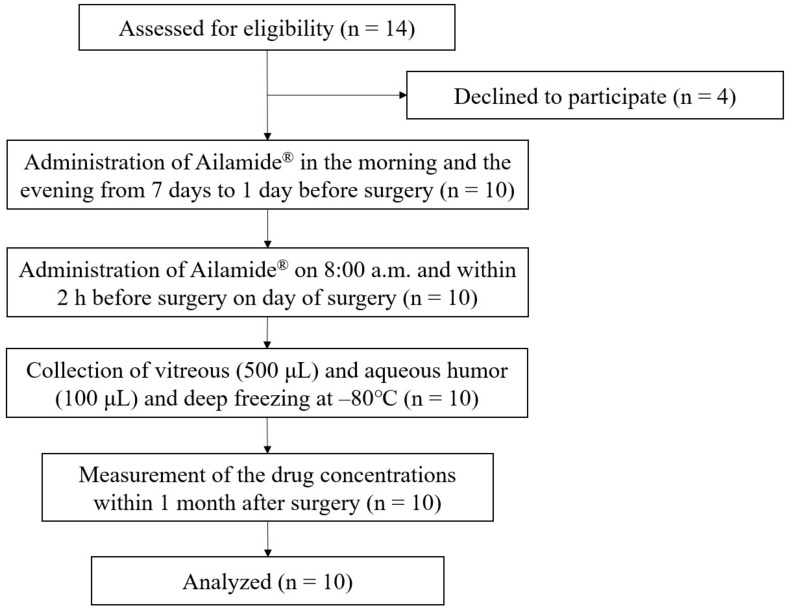
Study flow diagram.

Overall, 14 patients were enrolled in this study. Four patients withdrew their consent after agreeing to participate due to concerns regarding the increased number of eye drops and the complexity of the procedure. The usage of Ailamide^®^ (Senju Pharmaceutical Co., Ltd.) did not result in any adverse effects in those patients. The remaining 10 patients complied with the study protocol and were included in the analysis (Figure 1).

All patients (five males and five females) were Japanese, with a mean age of 67.7 ± 10.0 years (Table 1). Of the 10 eyes examined, nine and one eye were phakic and pseudophakic, respectively. Of the 10 patients, nine had an idiopathic epiretinal membrane. All patient data are available in the Supplementary Materials (Table S1).

All patients used levofloxacin ophthalmic solution 1.5% for 3 days preoperatively. Three patients had been using eye drops other than levofloxacin: pirenoxine ophthalmic suspension 0.005% and cyanocobalamin ophthalmic solution 0.02% (n = 1), pirenoxine ophthalmic suspension 0.005% (n = 1), and diquafosol sodium ophthalmic solution 3% (n = 1).

### 3.2. Primary Outcomes

**Figure 2:**
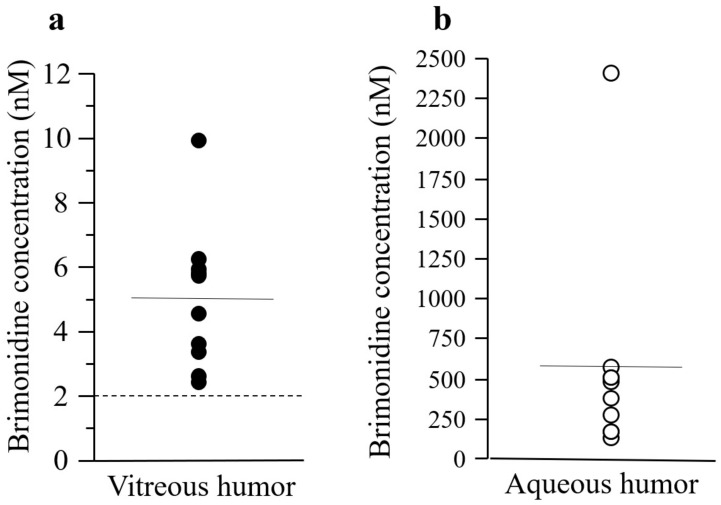
Concentration of brimonidine in vitreous (**a**) and aqueous (**b**) humor samples. Solid horizontal lines indicate the mean of the included data points. Dotted lines parallel to the x-axis denote the 2 nM concentration of brimonidine. Filled and open circles indicate the concentrations in vitreous and aqueous humor samples, respectively.

The mean concentration of brimonidine in vitreous and aqueous humor samples was 5.02 ± 2.24 (95% confidence interval [CI]: 3.41–6.62) and 559 ± 670 nM (95% CI: 79.9–1040), respectively (Figure 2). In all patients, the concentration of brimonidine in the vitreous humor was >2 nM.

**Figure 3:**
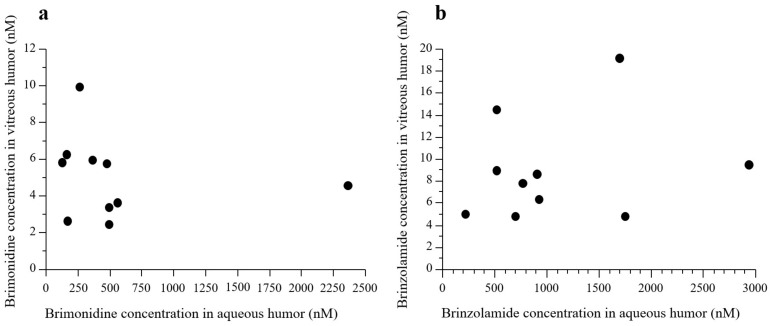
Correlations between the concentrations of drugs in aqueous and vitreous humor samples. There was no statistically significant correlation between the concentrations of brimonidine (**a**) and brinzolamide (**b**) in the aqueous and vitreous humor samples.

There was no statistically significant correlation observed between the concentrations of brimonidine in the aqueous and vitreous humor samples (*p* = 0.6567, R^2^ = 0.0259) (Figure 3a).

### 3.3. Secondary Outcomes

**Figure 4:**
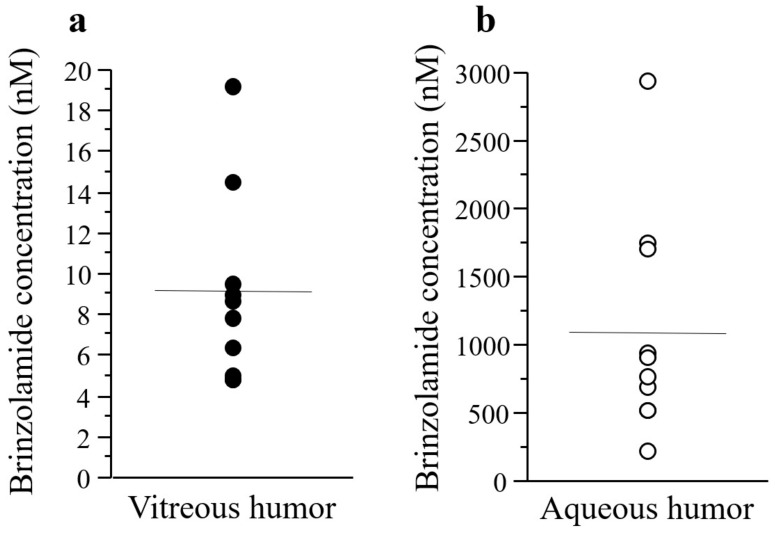
Concentration of brinzolamide in vitreous (**a**) and aqueous (**b**) humor samples. Solid horizontal lines indicate the mean of included data points. Filled and open circles indicate concentrations in the vitreous and aqueous humor samples, respectively.

There was no statistically significant correlation between the concentrations of brinzolamide in the aqueous and vitreous humor samples (*p* = 0.5479, R^2^ = 0.0469) (Figure 3b). The mean concentration of brinzolamide in vitreous and aqueous humor samples was 8.96 ± 4.65 (95% CI: 5.63–12.3) and 1100 ± 813 nM (95% CI: 513–1680), respectively (Figure 4).

**Figure 5:**
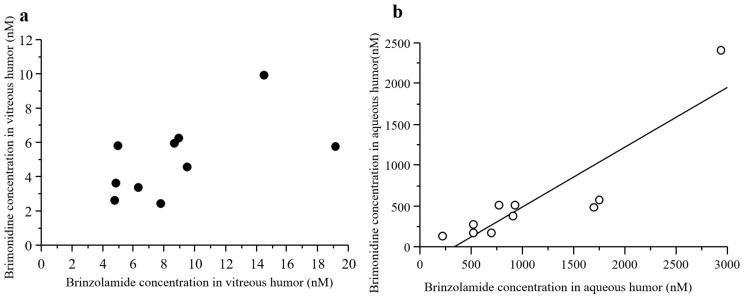
Linear correlations between the concentrations of brimonidine and brinzolamide in vitreous and aqueous humor samples. There were no significant correlations between the concentrations of brimonidine and brinzolamide in the vitreous humor samples (**a**). However, a significant positive correlation was noted in the aqueous humor samples (**b**). Filled and open circles indicate drug concentrations in the vitreous and aqueous humor samples, respectively.

There was no statistically significant correlation detected between the concentrations of brimonidine and brinzolamide in the vitreous humor samples (*p* = 0.0789, R^2^ = 0.336) (Figure 5a). Nevertheless, a significant positive correlation was recorded in the aqueous humor samples (*p* = 0.0006, R^2^ = 0.792) (Figure 5b).

Prior to the instillation, the mean IOP was 14.3 ± 1.9 mm Hg. The mean IOP prior to the operation was 11.2 ± 2.0 mm Hg, indicating a significant IOP reduction after the instillation (*p* = 0.0008; −3.1 ± 2.0 mmHg; 95% CI: −4.5–−1.7). Throughout the course of the study, there were no systemic or ocular side effects related to drug administration in any of the patients. Moreover, postoperative complications (e.g., infectious endophthalmitis, vitreous hemorrhage, and retinal detachment) did not occur in any of the patients.

The mean BCVA in logMAR prior to and 1 month following the operation were 0.41 ± 0.24 and 0.17 ± 0.25, respectively. The BCVA was significantly improved after surgery (*p* = 0.0009).

## 4. Discussion

Drugs must enter the vitreous cavity to exert their neuroprotective effects on RGCs [43]. Moreover, a concentration of brimonidine of >2 nM is needed in the animal retina for the activation of α2 adrenergic receptors in neuronal cells [36]. Therefore, the objective of this investigation was to determine whether the twice-daily topical administration of 0.1% brimonidine tartrate and 1% brinzolamide fixed-combination ophthalmic suspension (Ailamide^®^; Senju Pharmaceutical Co., Ltd.) for 1 week would result in a concentration of brimonidine > 2 nM. Following treatment, the concentration of brimonidine was >2 nM (mean concentration: 5.02 ± 2.24 nM; 95% CI: 3.41–6.62) in all vitreous samples.

Previous studies investigated the concentration of brimonidine in vitreous humor after the topical administration of 0.1% (Aifagan^®^, Senju Pharmaceutical Co., Ltd.) [33], 0.15% (Alphagan^®^; Allergan, Dublin, Ireland) [34] and 0.2% (Alphagan^®^; Allergan) [35] brimonidine tartrate ophthalmic solutions, and 0.1% brimonidine tartrate and 0.5% timolol fixed-combination ophthalmic solution (Aibeta^®^; Senju Pharmaceutical Co., Ltd.) [37]. Ailamide^®^ (Senju Pharmaceutical Co., Ltd.) is an ophthalmic suspension containing brinzolamide. The liquid properties of this formulation differ from those of other brimonidine tartrate-containing ophthalmic solutions. The present study is unique because the concentration of brimonidine in the vitreous humor was evaluated following the topical administration of an ophthalmic suspension.

Previous studies quantified the concentration of brimonidine in the vitreous humor following the instillation of ophthalmic solutions, including brimonidine tartrate. The investigators reported that the concentration of brimonidine in some samples was <2 nM. However, in the present study, the concentration of brimonidine was >2 nM in all vitreous humor samples. An experimental animal study also revealed that the concentration of brimonidine after the instillation of Ailamide^®^ (Senju Pharmaceutical Co., Ltd.) was equal to or higher than that of 0.1% brimonidine tartrate ophthalmic solution [44]. Differences in the composition of these formulations (e.g., viscosity) might contribute to the efficient penetration of brimonidine into the vitreous humor.

A significant positive correlation was detected between the concentrations of brimonidine and brinzolamide in the aqueous humor. Nevertheless, there was no significant correlation observed in the vitreous humor samples. Interestingly, in our previous study using Aibeta^®^ (Senju Pharmaceutical Co., Ltd.), we revealed significant positive correlations between the concentrations of brimonidine and timolol in vitreous and aqueous humor samples [37]. As brimonidine tartrate and timolol are clear solutions, they may have a similar ability to penetrate the vitreous humor. Furthermore, the suspension properties may affect the penetration of brinzolamide into the vitreous humor.

Consistent with previous studies [33,37], we did not find a statistically significant correlation between the concentrations of brimonidine in vitreous and aqueous humor samples. There results suggest that the routes of entry of brimonidine into the vitreous and anterior chambers differ. The results of experimental studies using rabbit [45] and monkey eyes [46] after topical instillation of radiolabeled nipradiol ophthalmic solution indicate that nipradiol is distributed in the posterior retina from the posterior periocular tissues across the posterior sclera. A previous study using rabbits investigated ocular drug delivery after topical instillation of brimonidine. The images obtained using matrix-assisted laser desorption/ionization imaging mass spectrometry suggest that the distribution of brimonidine to the posterior ocular region is distributed through the uveal-scleral pathway [47]. Additionally, we previously demonstrated that the use of 0.1% brimonidine ophthalmic solution did not result in a significant difference in the concentrations of brimonidine between phakic and pseudophakic vitreous samples [33]. These results support the hypothesis that brimonidine diffusion from the posterior periocular tissues to the vitreous chamber contributes to the neuroprotective effect of brimonidine ophthalmic solution in clinical trials [31]. Patients had been using eye drops other than Ailamide^®^ (Senju Pharmaceutical Co., Ltd.) The 10 min or longer interval between Ailamide^®^ (Senju Pharmaceutical Co., Ltd.) and another eye drop instillations was designed in the present study. Therefore, the possibility that brimonidine distribution in ocular tissues might be affected by another eye drop seemed to be minimized in this study.

To be deemed neuroprotective for retinal ganglion cells, the drug must meet the following four criteria: (1) receptors on its target tissues, such as the optic nerve or retina, (2) adequate penetration into the vitreous or retina at pharmacologic levels, (3) induction of intracellular changes that enhance neuronal resistance to insult or interrupt apoptosis in animal models, and (4) demonstration of similar efficacy in clinical trials [43]. As for the distribution of α2 receptors to which brimonidine binds, several studies using animal and human tissues showed the presence of α2 receptors in the retina and optic nerve head [48,49]. As for induction of intracellular changes, in vitro and in vivo experiments demonstrated that activation of α2 receptors by brimonidine upregulates the intrinsic cell survival signaling pathway and antiapoptotic genes including Bcl-2 and BCL-X_L_ [30]. Furthermore, many animal experimental models have shown that brimonidine offers neuroprotection in several types of ocular injuries, including retinal ischemia [50,51,52,53,54], optic nerve crush [29,55], photoreceptor degeneration [56], and ocular hypertension and glaucoma [57,58]. As for the demonstration of similar efficacy in clinical trials, clinical trials using brimonidine ophthalmic solution for patients with open-angle glaucoma [31,32] have shown that brimonidine attenuates the further progression of visual field loss rather than ß-blocker, as described above. Because our present study has shown that topical administration of Ailamide^®^ (Senju Pharmaceutical Co., Ltd.) delivered a concentration of brimonidine > 2 nM into the vitreous, it is possible that the fixed-combination ophthalmic suspension might also meet the four neuroprotective criteria.

The present study had several limitations. Firstly, we could not conclude whether brinzolamide in the vitreous humor following topical treatment with Ailamide^®^ (Senju Pharmaceutical Co., Ltd.) affects the posterior retina. Brinzolamide inhibits the carbonic anhydrase type II isozyme (CA-II) in the ciliary body, thereby reducing the production of aqueous humor [38]. Moreover, some clinical studies have reported that topical instillation of brinzolamide improves cystoid macular edema in uveitic eyes [59] and retinal blood circulation [60]. In the present study, the mean concentration of brinzolamide in vitreous humor was 8.96 nM; of note, this concentration was higher than the IC_50_ (3.2 nM) required for the inhibition of CA-II in the ciliary body [38]. Further in vitro and in vivo experimental studies are required to determine the optimal concentration of brinzolamide for inhibiting CA-II in the retina. Secondly, one patient had extremely high concentrations of brimonidine and brinzolamide in the aqueous humor, although the concentrations of these drugs in the vitreous humor were low. Corneal epithelial defects affect the penetration of ophthalmic solutions into the cornea [61]. However, underlying ocular disorders were not identified in these patients. Moreover, as the adherence in this study was self-reported, the high concentrations detected in the aqueous humor might have been related to overdose. Multicenter clinical study would be required to obtain further samples to evaluate drug concentrations of glaucoma vitreous.

## 5. Conclusions

Twice-daily topical instillation of a fixed-combination ophthalmic suspension containing 0.1% brimonidine tartrate and 1% brinzolamide for 7 days resulted in a concentration of brimonidine > 2 nM in all vitreous samples. However, there was no significant correlation observed between the concentrations of brimonidine in the vitreous and aqueous humor samples. Thus, another pathway to the vitreous chamber, different from the pathway to the aqueous humor, may be involved in the neuroprotective effects of brimonidine.

## Figures and Tables

**Table 1:** Patient characteristics.

Characteristic	Value
Total (n)	10
Age, mean ± standard deviation (years)	67.7 ± 10.0
Sex, male/female (n)	5/5
Lens status, phakia/pseudophakia (n)	9/1
Diagnosis	
Idiopathic epiretinal membrane (n)	9
Macular hole (n)	1

## Data Availability

The data presented in this study are available in the Supplementary Materials (Table S1).

## Supplementary Materials

The following supporting information can be downloaded at: https://www.mdpi.com/article/10.3390/jcm12134175/s1, Table S1: Patient data.
